# On the Origin, History and Treatment of Trichina Spiralis*A paper read before the Fox River Valley Med. Association, and published by request.

**Published:** 1870-02

**Authors:** 


A MONTHLY RECORD OF

*Medicine, Surgery, and the Collateral Sciences.*

EDITED BY J. ADAMS ALLEN, M.D., LL.D.; AND WALTER HAY, M.D.

VOL. XXVII. — FEBRUARY, 1870. — NO. 2.

### Original Communications.

### ARTICLE I. — *On the Origin, History, and Treatment of Trichina Spiralis.**

IN conformity to a usage established long ere this, I am before you, to-day, as your retiring presiding officer for the year just passed; and I beg your attention for a few moments, while I introduce some remarks upon the origin, history and treatment of a disease of which there is but little written and comparatively less known, in this our country, by the medical profession generally.

The disease to which I allude was nosologically arranged by Dr. Zenker, in 1860, is of parasitic origin, and has been called, by its discoverer, trichiniasis, from the name of the entozone producing it, the trichina spiralis. The generic portion of the name of this animal is derived from a Greek word, meaning or referring to a hair, in allusion to its filliform shape; the specific part, spiralis, from the Latin word spiralis, indicating the spiral watch-spring-like form which it assumes when curled within its cyst.

The trichina may very properly be described as a small, slim worm, measuring at adult age from $\frac{1}{60}$ to $\frac{1}{30}$ of an inch in length,

* A paper read before the Fox River Valley Med. Association, and published by request.

and from $\frac{1}{150}$ to $\frac{1}{100}$ of an inch thick, semi-transparent, with an attenuated end, which we shall describe as its head, the other or anal end being blunt.

When at rest, they usually assume a coiled form, but, when moving through the muscular tissue, their motion is spiral or cork-screw-like, with the attenuated end foremost, and are very active in their movements. From the mouth runs a tube to a sack, and from this runs a straight tube to the blunt end or anus. These seem to answer the threefold office of œsophagus, stomach, and intestine.

The females are much larger than the males. The ovaria and eggs of the females are inclosed in a long-tube lying alongside of the intestinal canal, and decreasing in size as it runs forward, and opening out at the beginning of the anterior third of the body.

The trichini, when found in its adult state, is usually inclosed in a thin, transparent capsule, lying between the sarcolemma of the primitive muscular fibre. This capsule, in process of time, frequently becomes calcareous, and in this rests the parasite for an indefinite space of time, unless sooner freed by entering the stomach of some living animal.

They are reproduced viviparously, and Virchow describes them as a bisexual parasite, he having found mature trichina, of both sexes, moving freely in the mucus of the intestines.

The first discovery of these little parasites was by Tiedeman, in 1822. Hilton, in 1832, found the pectoral muscles of an old man who died of cancer, thickly sown with minute white specks; an account of which he published in 1835 in the *London Medical Gazette*, and consequently was the first to put on record a description of what subsequently proved to be the cysts of trichina.

In 1833, James Paget, then a student at St. Bartholomew's, found what was supposed to be minute bony spicula, in dissecting a body, but on examining them with a microscope, he found each to be a cyst containing a small worm. As soon as he made known his discovery, portions of the muscles were distributed to various anatomists, and among others to Robert Owen.

In the same year, Professor Owen presented to the Zoölogical Society his memoir, containing a microscopic description of the entizone infesting the muscles of the human body; in which he describes the worm as destitute of intestinal canal and organs of generation, and classed them in a very inferior scale among the helminthes. In the same year, Dr. Farre discovered an intestinal tube and organs of generation, which placed the worm in a higher scale of organization, and enabled him to assign it a place among the class nematodes.

In the same year, or 1835, Henry Wood, of Bristol, gave to the *London Medical Gazette* the history of a case, which he diagnosed as an attack of acute rheumatism, complicated with cough and dyspnœa. The patient was a young man, aged 22; had been sick fourteen days, the last six of which he was confined to his bed; at which time he was admitted to the Bristol Infirmary, and died the tenth day after admission, or the twenty-fourth day from attack.

Autopsy exhibited pneumonia, extensive pericarditis, and trichina in the muscles of the chest und shoulders in great numbers. The trichina were not yet encysted.

In the same year, Dr. Harrison reported six cases of trichiniasis to the *Dublin Journal.*

In 1840, Kobett and Bischoff published an account of an autopsy made at Heidelberg, of a body infested with trichina. Bischoff and Farre mistook the attenuated end of the worm for its tail, in their description of it, and consequently described the mouth as the anus.

From 1842 to 1845, observations were published in this country, by Drs. Bowditch and Wyman, of Boston, and by Mouster and Soytzer, of Copenhagen, in Denmark.

In 1851, Suschka pointed out the attenuated filliform portion of the parasite as its head, and Leuckart, Bristowe and Rainey observed and demonstrated the genital organs, with other minute points of their anatomy.

In 1855, Zenker, Leuckart and Kuckinmeister experimented upon different animals, by feeding them trichinosed human flesh. Leuckart's observations were mostly made upon the dog, an animal not susceptible of trichinose infection; nevertheless he demonstrated the fact of the freeing of the trichina from their capsules, and their development to three times their primitive length, within three or four days after being taken into the stomach and intestines. He also established the fact that the worm would readily undergo gestation, and give birth to living young, even in the stomach and intestines of the dog.

The experiments of Leuckart fully demonstrated the fact that the young trichina commence their migration from the intestine towards the muscles, as soon as they were developed; which was proved to be about the eighth day after the food was ingested, and that, in the intestines of the hog, cat, rabbit, mouse, and domestic fowl, trichina would attain their full and complete sexual development.

Pagenstrucher and Fuchs devoted many months to artificial trichinization of various animals, viz.: Mammifers, birds, cold-blooded animals, fishes, molusks, and insects. Of the mammifers, the dog was the first subject. They found, as did Leuckart and Virchow, that trichina would propagate in the dog, but would not penetrate the intestines and enter the muscles. They found that the hog, wild-boar, guinea-pig, coney, hare, cat, rat, mouse, and mole were easily infected; while in the bull, ox and cow, the trichina would propagate, but, as in the dog, would not enter the muscle. The calf, goat, sheep, stag, and roebuck were moderately susceptible, and that badgers and martins remained doubtful.

The birds experimented upon were the goose, turkey, cock, hen, pigeon, starling, crow, jackdaw, jay, peacock and turkey-buzzard.

Although they found the parasite would become sexual and increase in them, yet they found them expelled by the alvine evacuations, and in the magpies no traces of trichina could be found four days after eating the meat. In the carp, frog, salamander, crab and flesh flies, the trichina were not developed at all. In the ducticus marginalis of the beetle family, trichina were found in the stomach alive five days after eating trichinosed meat. The observations of Pagenstecher and Fuchs are not fully confirmed by subsequent observations; for recently, in New York, Dr. Percy has reported a case in which a family were trichinosed by eating beef, and trichina were subsequently found in the beef.

Simon also cites two cases of persons having been trichinosed by eating beef in the epidemic of Calbe, and Kupprecht reports two cases at Hettstadt, and six in the epidemic at Leipsic, from eating raw beef.

If my memory serves me, Owen mentions having found trichina in oxen and pigeons that were fed on trichinose meat; the trichina were found in the neck, wing and thighs of the pigeons, eighteen days after feeding, thus showing pretty conclusively that the trichina will pass alive the ordeal (as a writer calls it) of the gizzard of the fowl, although by him denied.

It appears to be a well settled fact that many, if not all, of the ruminating animals are subject to become trichinosed if fed on food containing the parasite, but we should not expect them as good subjects as many other animals, from the fact of their stomachs containing usually a large amount of vegetable matter, in which the trichina might become intermixed, and consequently pass with the alvine evacuations without ever reaching the mucous membrane of the stomach.

In the case reported by Dr. Percy, of New York, it appears the animal was fed at a distillery — food, in our judgment, very proper to convey the trichina to the mucous membrane of the stomach of the animal, and also very favorable for the development of the sexual organs of the parasite.

Of the other cases reported of the trichina in beef, we have no means at present of knowing how the animals were fed.

In our description here given from the various authors, we are unable to trace the known origin of the trichina fifty years back.

Now, can we for a moment harbor the opinion that this little parasite has sprung into existence within the last fifty years. True, we have no authentic history of their existence for a longer time; but does this prove that they did not exist.

Until recently, we have been taught by geologists that there were no visible signs of animal existence below the lower silurian formation of rock; but recent discoveries in Canada have found the rizopods in the Laurentian formation some 30,000 feet below the lower silurian, the fact of their not having been discovered before, it would seem was no evidence of their non-existence. So we think it is with the trichina.

We have seen it stated, that Herod of old, the man-slayer and baby-killer, who died soon after the birth of Christ, died of trichiniasis.

The author making this assertion, cites Josephus as evidence of his position. In Josephus history, sixth chapter and fifth section, in speaking of Herod's disease, he says: "A fire glowed in him slowly, which did not so much appear to the touch outwardly as it augmented his pains inwardly; for it brought upon him a vehement appetite to eating, which he could not avoid to supply with one sort of food or other. His entrails were also exulcerated, and the chief violence of his pains lay in his colon; an aqueous and transparent liquor also had settled itself about his feet, and a like matter afflicted him at the bottom of his belly. Nay, further, his privy member was putrified, and produced worms; and when he sat upright, he had a difficulty of breathing which was very loathsome, on account of the stench of his breath and the quickness of its returns; he had also convulsions in all parts of his body, which increased his strength to an insufferable degree."

In this account of Herod's disease, given by Josephus, we find but very few, if any, symptoms which would lead us to believe his disease was that of trichiniasis. We would far sooner believe that he was troubled with a disease which attacks men in this day and age, even in our own country, who visit certain places where honest men ought not to go. At such places, they sometimes contract a disease which, if allowed to run, may cause their privy member to putrify and produce worms similar to those of Herod's, but not trichina.

Now, suppose Herod's system was infected with trichina, the worms found on his privy member were no evidence of the existence of that parasite.

The medical fraternity of that day and age had no means of seeing or detecting the parasite, as they could not be seen then any better than now, without the aid of lens; and the ancients of Herod's time had no such lens.

The first lens of which we have any account were made of rock crystals (probably quartz), in A.D. 50, and were found by Layard, in the palace of Nimrod. The microscope was not invented until the seventeenth century, consequently no such power could have been used, to detect trichina, in the days of Herod. Nevertheless we have reason for believing that trichina existed long before Herod's time.

In the eleventh chapter of Leviticus, the Lord spake unto Moses and Aaron, saying: "Speak to the children of Israel, that these are the beasts of which ye shall not eat, viz., camel, coney, hare and swine, because they are unclean."

This order was given to Moses and Aaron, in the year of the world 1490, or 2514 years before Christ; and it is remarkable that it should have included three of the most susceptible animals to the influence of the trichina yet known. And it is not wholly clear but what the first named (the camel) may yet prove to be easily trichinized.

Now, may not trichina have been the cause of this order then given to the Israelites, they being a people whom God took special care to look after and provide for, if we believe the account given of them in Holy Writ.

Genesis, first chapter and twenty-fifth verse, reads: "And God made the beast of the earth after his kind, and the cattle after their kind, and every thing that creepeth upon the earth after his kind; and God saw that it was good."

It appears that on the sixth day, God created every thing that creepeth upon the earth. Now, assuming this to be so, and also the fact that we have no account of any living being having been created since that day, have we not good reason for believing that the trichina was created at that time, although not discovered by man until the commencement of the nineteenth century.

Of their natural sphere and abiding place, as yet, we know nothing certain; but of their accidental appearance in man, and many of the lower class of animals, we have conclusive evidence; and that they will live for a succession of years in the muscular tissue of man, and many of the lower animals, when properly inclosed in their capsules, we have no doubt.

Scoutetten cites a case where the muscles of a subject who had been infected thirteen years previously, promptly developed and gave forth young upon being swallowed by a rabbit.

Virchow gives a case in which living trichina, within calcareous capsules, were found in a patient who had been trichinosed twenty-four years previously. Although this would seem to show that they are quite at home in the muscular tissue of man, yet we are constrained to say, as before, that they are here only by accident, and that neither man nor the swine are the natural abiding place of these little parasites. They are rarely found in either, and would, in all probability, become extinct if they depended upon either for their existence. Notwithstanding, they will readily propagate when introduced into the stomach of either.

Many cases of disease, produced by eating raw trichinosed meat, are reported in Germany, and among those most noted are the following:

At Plauen there were 40 cases, and one death; Magdeburg, Newstadt and Buckaw, about 400, with no deaths reported; Blankenbourg, 78, with one death; Calbe, 38, and eight deaths; Hettstadt, 159, with 28 deaths; and at Hedersleben, 350 cases, with 80 deaths; making, in those few epidemics, one thousand and sixty-five cases, with one hundred and eighteen deaths, or one death in nine and a fraction over.

As among the cases occurring in this country, we would cite the following:

That of a German family, reported by Dr. Schnetter, of New York city, in 1867, in which a whole family were affected, one of whom died. They had been eating raw ham, which, on a microscopic examination, was found to contain trichina. Dr. Voss also reports three cases on board of a Bremen ship lying in New York harbor. To satisfy himself of the existence of trichina, he harpooned the deltoid muscle of the mate, in which trichina was found, and an estimate made of about 7,000 to the cubic inch.

Dr. Lathrop, of Buffalo, has reported a case, and Dr. Horr, of Dubuque, Iowa, has reported six cases, as occurring in Mane township, in 1866 — that of three boys and three girls, who, on the 22nd of April, took a lunch of sandwiches, in the construction of which raw smoked ham was used. Two days after, all but one of them were attacked with diarrhœa, developing into all the symptoms of trichiniasis. Effective cathartics were given at once, which, doubtless, expelled the greater portion of the worms before they had time to multiply fatally. One subject, a girl of sixteen years, exhibited the symptoms in reversed order — lameness of the muscles on the second, and the diarrhœa on the third day. She was more severely ill than the others; had inflammation of the lungs and hoarseness of the voice; and, on the 19th of June, was hardly able to walk. No cathartic was given to her, as to the others. Dr. Horr also reports nine cases in Marion, in the family of Mr. Bemis; they were all taken ill about the first of May, after eating freely, at several times, of raw smoked ham, from four to ten days previously. Mr. Lansing ate freely from the same ham, well cooked, and showed no symptoms of the disease. The symptoms of this disease as shown in these cases, were, diarrhœa on the second day; pain, soreness, and debility of the muscles of the limbs, on the third day, with swelling of the face; followed, on the fourth and fifth days, by swelling of the hands, then of the feet and legs, with fever of a low grade, quick pulse, furred tongue, and sleeplessness. The muscular soreness and pain, with fever thirst, tenderness of the abdomen, and profuse sweating, continued as the main symptoms, until death, in the second or third week, or convalescence, slowly established, in from four to eight weeks. Two of the fatal cases had serious pneumonic complication. Five of these nine cases proved fatal: A little boy, June 1; H. B. on the 3rd; a child on the 8th; Mr. B. on the 15th; and Mrs. B. on the 17th. Examination after death, on two of the bodies, showed the parasite in the muscles, estimated by others, and myself, at about 200,000 per cubic inch. Some were also found in the lungs and spleen. We have given the above cases reported by Dr. Horr almost verbatim, for the purpose of comparing the symptoms with those of which we are now about to report, of the family of Mr. T. W. Tefft, of Elgin township:

On or about the 18th of February, 1869, Mr. T. was taken with a diarrhœa, and was not able to be at his work, but did not think himself sick enough to call a physician. The bowel complaint continued about the same until the first of March, at which time it became much augmented; and at this time two of his children were taken sick, and a day or two subsequently his wife was taken.

Being absent from home, we did not see the cases until March 11, at which time we learned that, when first taken, the three last cases were slightly sick at the stomach, but did not vomit, with general febrile symptoms, and occasionally a sharp, cutting pain in the stomach and upper portion of the bowels. Mr. T. had a partially dry and very red tongue, with a desire to wet it every few minutes; skin dry and hot by turns, and then drenched with sweat; pulse 140 per minute, small, with artery quite firmly contracted on circulating fluid; bowels discharging frequently a copious liquid, light-colored, horrid-smelling evacuation. He had a sharp pain in the left side, directly over the attachment of the diaphragm to the side, with cough, and an inability to lie upon the affected side; was wakeful, without any aberration of mind. There was more or less tenderness over the abdomen and also of the muscular system generally. He had a voracious appetite to eat, and desired the strongest kind of food, like pork, cabbage and potatoes, of which he would make a good meal for a well man, although, at the same time, his tongue was as red as a piece of beef. He was emaciated, and the countenance assumed a contracted, anxious appearance.

The medicine prescribed at this time consisted of pill hyd., grs. 3, pulv. gm. opii, gr. 1; one to be taken every eight hours, alternated with spts. turpentine, gtts. 25, in simple syrup. The blue mass was given to change the secretions and appearance of the evacuations of the bowels. The pill appeared to have no effect in changing the appearance, or controlling the evacuations, and after using it for two or three days, its further use was discontinued, and he was put upon muriatic acid, in 10-drop doses, well diluted with sugar and water, for the purpose of checking the profuse perspiration. After using this for about 48 hours, the sweating very much abated, and it was discontinued, and he was then put upon a solution of nitras argenti, the strength of which was half of a grain to the teaspoonful of rain water, to be largely diluted with water before taking. Of this solution he commenced with one-half a teaspoonful every six hours, which was increased to one, and finally to two teaspoonfuls at a time. This had the happy effect of checking the bowel complaint, and he began to amend, but his convalescence was very slow and protracted for months.

Now, while we firmly believe that the case of Mr. T. was one of trichiniasis, yet we are of the opinion that his being unwell, with a bowel complaint, at the time of eating the meat, which undoubtedly was raw, and in small quantity, and the increased irritation of the stomach and bowels, caused by the presence of the trichina in them, with the frequent evacuations of the same, thereby removing very many of the young trichina as soon as produced, aided him very much. That trichina passed through the coats of the bowels and entered the muscles in his case, we have no doubt; but far less in number than in the other cases of his family.

Mrs. T., as we have before stated, when first seen was laboring under general febrile symptoms; the tongue was nearly covered with a light-brown coat, the edges and tip of which were red, and the same redness extended a little way down the centre; the bowels were constipated; urine scanty. She had but very little pain, and that little was confined to the epigastric region. She also had a slight, hacking cough, without expectoration; the face was deathly pale, alternating with a very slight crimson flush, apparently deep-seated in the dermoid tissue; the pulse was from 150 to 160 per minute, small, with the artery quite firmly contracted on the circulating fluid. There was a frequent desire for cold water, but the amount taken at each time was small, it seeming that there was no great desire for very large potations.

As with her, so it was with the children, the symptoms up to the 11th of March being very similar in the three cases, the little boy apparently the sickest of the family. The brain did not seem to suffer much, all being perfectly sane, but a sort of wakefulness was plainly visible in each. From their appearance, we judged the exciting cause, whatever it might be, to be the same in these three cases, and the like was presumed to be the case with Mr. T., although, in some respects, he was somewhat different from the others. At our second visit Dr. Winchester was invited to visit the family with us, and kindly accepted the invitation.

The possibility of the family being affected with trichiniasis was then and there thought of, and all the facts then accessible were there elicited, and we were forced to the conclusion that this must be the disease of which they were afflicted. In coming to this conclusion we had many obstacles to surmount. First: The hogs from which the pork was made were reared on the farm, in a yard of about an acre; were out of this but once or twice, and then for but a few moments; were sixteen months old, and never known to be sick; they were butchered about the 1st of December, 1868, were well fattened, and the pork looked nice; the family had eaten of it from that time up to about the time of sickening without any bad effect; they had no recollection of eating any of it raw.

Previously, the house had been searched from garret to cellar, and the well from which they used water had been cleaned, without finding any local cause for the sickness of the family.

At this time the exciting cause, whatever it might be, lay hidden from view, and we only had the symptoms, as they then appeared, to guide us in our diagnosis. As the disease traveled on, the symptoms became more thoroughly developed, and the opinion before advanced was now much strengthened. A day or two subsequent to this consultation, a portion of the ham then in use by the balance of the family was examined under the microscope, and gave no evidence of trichina. On the 16th, the face of the wife and children began to swell about the eyes, and the muscles generally through the system began to be sore to the touch, which continued to increase; and on the 18th of March the muscles of the face and limbs evidently began to waste and the feet to swell. The œdema of the feet did not extend up the limb above the ankle at any time during the sickness of either of the patients. At this time there was a hoarseness of the voice and difficulty of swallowing. The patients were on the bed, flat upon their backs, without the power to move, or the ability to be moved, without suffering the most excruciating pain.

The bowels were still constipated, the abdomen was tympanitic and extremely tender to the touch; the pulse was decidedly quick, the skin at times a little warmer than in health, and at other times covered with a profuse perspiration. They now complained of great debility, but no pain unless moved. The appetite was good, with a desire for the heartiest kind of food. The urine was scanty, pale, and in one of the cases was for a time passed involuntarily.

On the 20th of March, and up to the 28th, there appeared to be some abatement in symptoms; but on the 29th they began to increase in severity, and continued so to do with Mrs. T. until the 6th of April, when death closed the scene with her.

The little girl began to amend about this time, but the little boy continued very sick until about the 20th of April, at which time he began to improve, but his convalescence was slow and tedious, it being some three months and a half from the attack before he could walk, and then for a time he could not get his heel to the floor.

The treatment advised in these three last cases was, first, a cathartic, composed of 10 grs. submuriate hyd., 15 grs. conv. jalapa, 1 gr. podophyllin, and 1 gr. c. ipec. for the mother, and two-thirds of a like dose for each of the children, to be followed in six hours with oil and turpentine if the bowels were not freely moved. After the bowels were freely opened, they were each put upon an emulsion of spts. turpentine, in doses of 20 drops of turpentine for Mrs. T., and from 10 to 15 drops for each of the children, to be given every four hours.

While using the emulsion, they were allowed a liberal diet of mutton or chicken broth and condensed beef, with rice and crackers. A free use of the chinchona bark, together with other tonic medicines, were substituted in a few days for the turpentine emulsion.

Mrs. T. continued from the 11th day of March without any decided change up to 1st April, at which time she began to complain of great prostration, attended with a frequent, slight cough, with but very little expectoration, but a marked hoarseness was plainly discernable. On the 3rd, 4th and 5th of April, she still complained of the great prostration, with the remark that it seemed to her that she had not the power to fill her lungs with air, but was in no pain when lying still, but in great pain when moved. She also remarked that it appeared to her that she would fall to pieces if lifted from the bed on which she was then lying. A subsequent examination of the body demonstrated the fact that the muscular fibre was almost wholly destroyed, explaining without doubt the cause of those feelings.

Of the cases reported by Dr. Horr, in the main, the symptoms are much like those of Mr. Tefft's family, with this difference: his cases had diarrhœa on the second day, while three of those we attended had no diarrhœa at any stage, and the fourth one had a diarrhœa for some two weeks before being taken down with the disease. Then, again, his had pain, soreness and debility of the muscles of the limbs on the third, with swelling of the face, followed on the fourth and fifth days by swelling of the hands, then of the feet and legs; while in those we attended the soreness of the muscles did not commence until the twelfth day, and they had no pain unless moved; the face bloated on or about the fifteenth day, and a day or two subsequently the feet swelled, but the legs did not bloat at all, but rapidly emaciated. In the little boy, the scrotum became swollen about this time, which lasted for a few days only. Mrs. T. died on the thirty-fourth day of her sickness, the time of death from attack differing but few days from that of the Bemis family.

One of the cases reported by Dr. H. was that of a young lady, who, he says, had lameness of the muscles on the second, and diarrhœa on the third day. Now we are disposed to doubt the ability of trichina to enter the muscles so soon after the ingesta of the pork, and we cannot see how they should produce their specific effect on the muscle before they enter it. We here assume the ground that no parent trichina ever enter the muscles a second time. When taken into the stomach, they must necessarily occupy from four to six or eight days in being liberated from their capsule, conceiving, maturing and depositing their young, which, when so deposited, are the trichina which in their turn enter the muscular system. Now, if this be correct, we would not expect any pain or soreness of the muscles for the first six or eight days after the ingesta of the meat. It also appears to us that there must be a second brood of these little parasites, which are developed on or about the twentieth day after the ingesta of the meat, perhaps a little later. Our reasons for this opinion are simply these: First, each of the patients under our care was taken much worse on or about the twenty-eighth day of the disease, and it was not until this second attack (if so it may be called) that we seriously feared that Mrs. T. would not recover. From the soreness and emaciation of the muscles, we had every reason to believe that the trichina was there at work long ere this; and, furthermore, on examination of the body of Mrs. T., made on the twenty-fifth day after death, trichina were found encysted, and others not fully grown and of course not yet encysted. Now, had they all been produced at one and the same time, we should have expected them all to have arrived at maturity at or near the same time.

Trichiniasis is one of the most dangerous of all parasitic diseases, and it behooves the physician to study well the nature, origin and development of the entizone producing it, as well as the early symptoms of the disease.

The effectual treatment of the disease is confined to a few of the first days of the sickness of the patient, and before the parasite has time to mature its young, which it will undoubtedly do within eight or ten days after the ingesta of the meat. When once the trichina have wormed their way through the coats of the intestines, they are then beyond the reach of medicine, and we have only to wait for them to feed, mature and encyst, which, if our patient has the good luck to survive, will most assuredly recover as far as is consistent with the amount of these little parasites left imbedded in the muscular tissue, which he will undoubtedly ever have to carry through life.

If this be correct, then how important to diagnose the disease at an early day, and apply without delay the only treatment which, in our judgment, has any chance of mitigating the evil effects of this little parasite.

We would commence our treatment at an early day, with the free use of active cathartics, followed immediately with a liberal use of the turpentine emulsion. Prof. Mosler, of Berlin, advises cathartics, followed with the following mixture, to destroy the trichina while in the intestines ; benzine, 2 ʒ, muc. of gum arabic, liquorice juice, each 1 ℥, in 4 ℥ peppermint water ; of this a tablespoonful is to be given every one or two hours. The carbolic acid has also been advised, with a view of expelling the trichina while still in the intestines.

Prof. Fredrichs recommends the picronitrate of potassa as a remedy, to be given in grain doses, to remove the trichina from the system after they have entered the muscles, and cites a case as evidence of its good effect, where he harpooned the muscles several times, the last of which showed no trichina. The power of removing the trichina from the muscles by the use of the pichronitrate of potassa, as claimed by Prof. F., has not been confirmed by subsequent observations ; and allow us here to reiterate what we have before said, that we do not believe that any known medicine possesses the power of removing the trichina when once encysted in the muscular tissue.

Therefore, we most emphatically say that, in our opinion, the first few days is the only time in which we can have any hopes of removing trichina from the system, and if this be passed without proper means having been employed, we may fold our arms and be lookers-on while our patients are being tossed about upon the turbulent sea of disease, without the means of rendering any, or but very little, real assistance, at most. We would here repeat and earnestly enjoin the old Scotch physician's recommendation of two important things to be done by his patients, in order to the enjoyment of good health ; the first of which was, to always keep the fear of the LORD before your eyes ; and the second was, to always keep your bowels freely open with cathartics.

On this last hangs the sheet-anchor of the early treatment of trichiniasis, in our judgment.

In conclusion, allow us here to proffer our many thanks to Col. Beatty, of Elgin, for his kindness in first detecting the trichina in the pork of Mr. Tefft. He being a microscopist of much experience, we took some of the suspected meat to him, and he soon demonstrated the trichina to us, in great numbers, since which time we have personally examined much pork and many hogs, both in this State and Minnesota, and have not been able to detect the trichina in a single instance, other than that of the pork of Mr. T.

In the *Medical Examiner*, for May, 1866, we find the report of a committee, made to the Chicago Academy of Sciences, in which they state that, of 1394 hogs examined, they found trichina in 28, making one in 50, or very nearly so ; while in Brunswick, of 19,747 hogs examined, there were but two found containing trichina, or one in 10,000, as against 1 in 50 in this country. The comparative immunity from the disease in our people results, no doubt, from the thoroughly cooking of the meat before eating it, while in Germany much is eaten raw.

According to the best information we are able to obtain at this time, it will require about 190° Fahrenheit, of dry heat, to effectually destroy the parasite.

It has been stated that 106° Fahr. would destroy the trichina most assuredly. This is only eight degrees above blood heat, and it would appear to us that the statement is an error. Now, one who is at all conversant with the tenacity with which this little parasite holds to life, would argue or attempt to state that so low a temperature as 106° Fahr., would effectually destroy the parasite, or securely protect the eater of meat containing them.

A cubic inch of meat containing trichina has been boiled for twenty minutes, and a portion fed to a rabbit, which was trichinosed ; but a portion of the same meat was boiled a few minutes longer and fed to another rabbit, which produced no effect on it.

It has also been said that a saturated solution of chloride of soda would kill this parasite in a few minutes, and that meat, salted and smoked, would wholly destroy the parasite. This, most assuredly, is not so; for the ham eaten by Mr. Tefft's family was both well salted and smoked before eating. Therefore, we are constrained to say that, in our judgment, the only safety in eating pork is in having it well and thoroughly cooked.

Since penning the foregoing, we have received, through the kindness of Dr. Reed, of Hampshire, a specimen of pork and sausage, from a family living in the western part of Kane county, who were taken dangerously ill after eating of the meat, a part of whom have since died.

This meat and sausage, according to our estimate, contains about 8000 trichina to the cubic inch.

In regard to the history of these cases, we know but very little that is reliable. But we know sufficient to induce us, again, to urge upon the medical profession the importance of thoroughly posting themselves in the early history and symptoms of this much-to-be-feared disease, so as to be liable to diagnose it at sight; for on this and the early judicious treatment, may and undoubtedly will depend the life of our patients, in very many cases.

While trichiniasis was only known to Germany, our people had but little fear of the disease; but now that it has become a disease known to exist this side of the Atlantic, and even to be laid at the threshold of many of our doors; and when we come to reflect that, according to the report of the committee at Chicago, made in the year 1866, of hogs sold in that city, we are liable to the disease (all other things being equal) as 200 is to 1; or, in other words, 200 hogs are trichinosed here to 1 in the German States, where it has prevailed to such an alarming extent.

In view of these facts, it would seem that our people should awaken to the reality of our situation, and take measures to devise, if possible, some means to protect the people of this country from the ravages of this much-to-be-feared disease.

